# A Review on Methods of Risk Adjustment and their Use in Integrated Healthcare Systems

**DOI:** 10.5334/ijic.2500

**Published:** 2016-10-26

**Authors:** Christin Juhnke, Susanne Bethge, Axel C. Mühlbacher

**Affiliations:** IGM Institute Health Economics and Healthcare Management, Hochschule Neubrandenburg, Neubrandenburg, Germany; Institute of Epidemiology, Social Medicine and Health System Research, Hannover Medical School, Hannover, Germany

**Keywords:** Risk Adjustment, Integrated Care, Evaluation

## Abstract

**Introduction:**

Effective risk adjustment is an aspect that is more and more given weight on the background of competitive health insurance systems and vital healthcare systems.

The objective of this review was to obtain an overview of existing models of risk adjustment as well as on crucial weights in risk adjustment. Moreover, the predictive performance of selected methods in international healthcare systems should be analysed.

**Theory and methods:**

A comprehensive, systematic literature review on methods of risk adjustment was conducted in terms of an encompassing, interdisciplinary examination of the related disciplines.

**Results:**

In general, several distinctions can be made: in terms of risk horizons, in terms of risk factors or in terms of the combination of indicators included. Within these, another differentiation by three levels seems reasonable: methods based on mortality risks, methods based on morbidity risks as well as those based on information on (self-reported) health status.

**Conclusions and discussion:**

After the final examination of different methods of risk adjustment it was shown that the methodology used to adjust risks varies. The models differ greatly in terms of their included morbidity indicators. The findings of this review can be used in the evaluation of integrated healthcare delivery systems and can be integrated into quality- and patient-oriented reimbursement of care providers in the design of healthcare contracts.

## Introduction: Risk Adjustment

Effective risk adjustment is an aspect that is more and more given weight on the background of competitive health insurance systems and vital healthcare systems [1].

The objective of risk adjustment is to generate and provide information about the risks of morbidity and the risk factors within a specific population group. Based on the obtained risk structure the expected utilisation as well as its costs in future periods shall be predicted. The risk structure of the providers plays a vital role in Pay for Performance (P4P). A prerequisite for optimal incentive-based service models is a (partial) dependence of the agent’s returns on the provider’s gain level. The risk presented within a population and indicator oriented contracting must be measured and risk adjustment conducted.

In the last 20 years several methods of risk adjustment have been developed, especially in the United States. Common to most of these models is the fact that patients/beneficiaries are assigned to homogenous risk groups according to their diseases and their need for care on the basis of routinely available data on the utilisation and diagnosis of hospitals and outpatient care.

The aim of using such morbidity-oriented classification methods is to achieve a reliable and robust quantification of the expected (present or future) resource utilisation. In terms of their selectivity, these methods are far superior to the “previous” methods which are based only on the demographic characteristics of age and gender and possibly the status of reduced earning capacity [2].

Based on this knowledge four main research questions are defined that should be answered:

1. “Which risk factors can be used or are currently used in risk adjustment?”
2. “How can these risk factors be combined?”
3. “Which methods of risk adjustment are currently used in international healthcare systems to adjust risks and to predict cost as well as utilisation?”
4. “Which special characteristics do these methods of risk adjustment currently used have and how can they be compared to each other?”

Accordingly, the objective of this review was to obtain an overview of existing models of risk adjustment as well as on crucial outcome parameters and weights in risk adjustment. Moreover, the predictive performance of selected methods in international healthcare systems was analysed.

## Methods: Literature review

Numerous methods are used for the risk adjustment in several health systems. A comprehensive, systematic literature review on methods of risk adjustment was conducted in late 2012 in terms of an encompassing, interdisciplinary examination of the related disciplines. The review was based on an initial search in the databases PubMed/Medline as well as Cochrane Library.

Additionally desk research included the search in the databases of Web of Science, The Commonwealth Fund, World Health Organization (WHO), Agency for Healthcare Research and Quality (AHRQ), and the International Society for Pharmacoeconomics and Outcomes Research (ISPOR).

**Figure 1:**
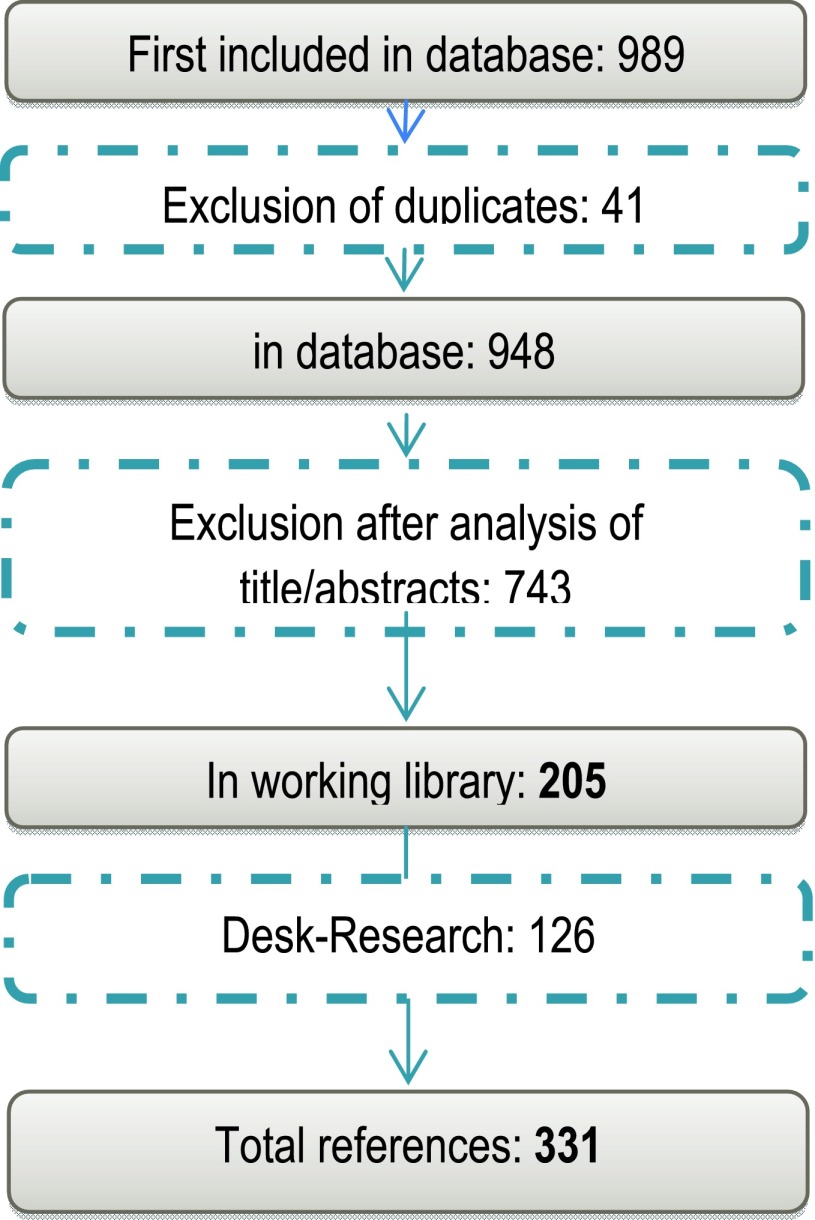
Systematic Literature Research.

Overall the literature search resulted in 34,072,869 hits. By combining the most relevant search terms the search could be narrowed so that in a first step 989 references have been imported into the first library. (Figure 1)

In the second step these references underwent an analysis of title and/or abstracts. Doing this the research group used predefined inclusion and exclusion criteria. These included language (= German, English), publication date (> 1980), and content (calculation of insurance premiums and fees, review article of certain methods of risk adjustment, method comparisons). Excluded were articles that dealt exclusively with therapies of different diseases/illnesses and/or clinical trials, quality management, statistics, quality of life (not related to risk adjustment) as well as duplicates. After analysing abstracts and full texts and together with an ongoing desk research 331 references could be extracted.

## Results

In general, a thematic distinction could be made, since the articles included in this review addressed different fields in medicine and care, such as pediatrics, intensive care, geriatric care or long-term care. Moreover risk adjustment methods in widespread diseases, e.g. cardiovascular diseases, or chronic diseases as well as different healthcare delivery systems (managed care, disease management, and integrated care) were analysed. Finally a distinction can be made in terms of specific indications and/or treatments, such as fall prevention, cancer, severe injuries. Accordingly, a distinction between the healthcare systems targeted in the reviewed articles is possible. The articles included descriptions on risk adjustment methods in several countries, e.g. Germany [3,4,5,6], Australia [7,8,9,10], Switzerland [11,12], Hungary [13], USA (Medicare [14,15,16,17,18,19,20,21], Medicaid [22,23,24,25], Veterans Affairs [26,27,28,29,30,31]), Spain [32,33], Sweden [34,35,36], Canada [37,38,39], The Netherlands [40,41,42,43], Taiwan [44,45,46,47], Chile [48,49], Israel [42,50], Great Britain [41], Italy [51], Poland [52], and Bulgaria [53].

### Research Question: Risk factors

The basic principle of risk adjustment is to identify the crucial health risks, and to compare the various groups of insured persons, to forecast their future expenditures for health services [54].

Risk factors can be identified on the basis of different information. Therefore the first question that should be answered with the help of the literature review was “Which risk factors can be used or are currently used in risk adjustment?”

Looking at different methods of risk adjustment it becomes clear that the models differ greatly in terms of morbidity indicators included. However, the same basic indicators/risk factors such as *age* and *gender* are included for risk adjustment within most models. The *diagnoses*, both inpatient and/or outpatient, are recorded for many models such as the Adjusted Clinical Group, the Chronic Illness and Disability Payment System, the Diagnostic Cost Group and RxGroup, but also in the German Hierarchical Morbidity Groups (HMG)-method. This risk factor often also includes the *severity of the disease* that accounts for different risk categories. Another common risk factor often considered is *prescription of drugs*. It is included, e.g. in the RxGroup, the Medicaid Risk Group, the Rx risk, and the Pharmacy cost group. The *disability statuses* as well as the *employment status* are other risk factors that are included in several methods. The German HMG-model, a mortality-based risk adjustment scheme for the Social Health Insurance (SHI) also incorporates the “*entitlement for sickness allowances*”.

As most of the methods considered in this overview were developed in the US the “*Medicaid/*Medicare eligibility” of a person is another indicator often considered since many models were specifically developed for these organizations.

The question which risk factors are included in the determination of resource requirements depends on the specific model of calculation [55]. Risk factors can arise on the basis of personal information, e.g. age, gender, income or profession. However, it is essential to consider also information on utilisation of services, diagnoses or the medication needs of the insured, and references to the self-assessment of the patient, e.g. general health or (health-related) quality of life [56].

The information needed for risk adjustment can be categorised in different ways. One possible categorisation is the following:

- Demographic characteristics, e.g. age, gender, origin, ethnic group
- Clinical factors, e.g. diagnoses and comorbidities
- Socio-economic characteristics, e.g. education, income or marital status
- Health behaviours, e.g. smoking, alcohol consumption and diet
- Preferences concerning quality of life and expectations on the healthcare system [54]

### Research Question: Combination of risk factors

After identifying the various risk factors, the insured patients need to be assigned to certain classes in order to evaluate them. Depending on the respective evaluation and calculation model used, existing comorbidities will be incorporated in different ways.

The second question of interest was “Which combination of risk factors is used?” This includes the question of which methods of risk adjustment are currently used in international healthcare systems to adjust risks and to predict cost as well as utilisation.

Looking at the results of the literature review it seems to be reasonable to make a differentiation by three levels:

1. Risk adjustment methods based on mortality risks
2. Risk adjustment methods based on morbidity risks, either based on pharmaceutical information or diagnoses
3. Risk adjustment with information on (self-reported) health status

Another commonly used distinction is made between the cell and the regression approach. In the case of the cells approach the patient is classified according to his or her health factors and morbidities to exactly one group. Based on the information on the number of insured persons in one group and the average treatment costs, the average per capita costs for each group are calculated. Thus, the various groups of insured are easier to compare. By contrast, in the regression approach, a basic capitation fee, which depends, e.g. on the age and gender, is added with an extra factor depending on the individual risk factors of the individual. Thus, a provider receives more surcharges for the treatment of a patient with many risk factors than for a patient with fewer risks. The total amount of reimbursement is therefore the sum of all individual compensations [56,57,58].

#### Risk adjustment methods based on mortality risks

The mortality rate is considered to be the most important quality indicator. However, the mortality rate of a hospital depends not only on the quality of the services provided, but is influenced by pre-existing conditions, varying degrees of severity of underlying diseases and disease-independent characteristics such as age or gender. Thus, the clinical and economic outcomes of a hospital also depend on the risk profiles of its patients. A hospital treating high-risk patients very often has a higher mortality than a hospital with few high-risk patients. A worse outcome in such cases does not automatically imply a poorer quality of care. Only a reasonable consideration of varying risks in the patient population/case mix ensures a fair comparison and may prevent prejudices in terms of referral patterns, reluctance to operate on high-risk patients. Moreover it may affect resource allocation [59]. For this purpose risk-adjusted quality representations are used. In order to recognize and weight a possible common influence of multiple risk factors logistic regression models can be used.

Well-known examples of mortality-based indices are the Cardiac Surgery Reports [60,61], Charlson Comorbidity Index [54] or the Acute Physiology Age Chronic Health Evaluation (APACHE) [62].

It is widely accepted that costs have to be considered in healthcare decision-making. The future discussion will focus on the quality of the treatments and the ways this quality can be assessed and measured. In surgery operative or hospital mortality is mostly seen as the prime quality indicator [63]. However, the pure consideration of mortality is a rather weak measure of quality when it comes to variations in the case mix of patients. If operative mortality shall be used as quality measure and risk adjustment tool it has to be adjusted to the underlying case mix of a hospital [64].

#### Risk adjustment methods based on morbidity risks

In general, a morbidity-oriented classification is not a clinical but rather a cost-based classification.

Unlike so-called episodic-based models that are used for the calculation of per-case flat rates for hospital discharges (e.g., Diagnosis Related Groups, DRGs), classification models for beneficiaries are usually person-oriented methods. This includes that the classification is based on morbidity data for a full year. Moreover, resource utilisation is calculated and forecasted for a defined period of time (one year). Hence, estimated expenses are not oriented on a certain care period (as in DRGs) but rather encompass the full range of healthcare services and providers [2].

In general, classification models can be subdivided into models using only information from one care sector, e.g. outpatient medication prescriptions, and those models using the same indicators from different care sectors, e.g., inpatient and outpatient diagnoses. These models are also called *integrated models* [2] and sometimes referred to as “*all-encounter”* models, as they add information from inpatient encounter records to outpatient encounters [65,66].

#### Risk adjustment methods based on pharmaceutical information

Pharmaceutical information can be seen as an indicator of chronic diseases [58]. In order to use this information, the active agents are assigned to diseases typically treated with the help of that drug [67]. As the Anatomical Therapeutic Chemical Classification System with Defined Daily Doses (ATC/DDD) -system by the World Health Organization [68] provides a unique classification of therapeutic drugs the international comparability is facilitated.

An advantage of the usage of pharmaceutical information is its complete availability. Data on prescriptions are often available even when diagnostic data is missing [22]. Moreover pharmaceutical information is hardly manipulable, compared to other data [69]. Internationally several methods are available. In order to compare the different methods with each other a list of certain characteristics has been defined (developing institution, objectives, cell vs. regression approach, time of calculation, etc.). As far as possible the analysed methods were assessed in terms of these. Table 1 gives an overview on currently available methods as well as those used in past years [55,58,65,66,67].

**Table 1:** Risk adjustment methods based on pharmaceutical information.

Method	Cell Approach vs. Regression Model	Risk Factors used	Development Objective	Developing Institution	Developed by (primary author)	Calculation	All-encounter Model	No. of Groups	Comorbidities included	Grouping	Clinical meaningful/interpretable

**CDS**	Aggregate model (Regression)	Age, Gender, Drug prescriptions	Measurement of chronic disease status	Center of Health Studies, Group Health Cooperative of Puget Sound, Seattle, WA, USA	Van Korff et al. [71] Clark et al. [70]	Prospective	No	28	Additive weights	Incomplete	Yes
**Medicaid Rx**	Aggregate model (Regression)	Age, Gender, Drug prescriptions for certain conditions	Development of risk-adjusted reimbursement/compensation systems for Medicaid	University of California, San Diego, CA, USA	Gilmer, Kronick, Dreifuß [22]	Prospective	No	45/48	Considered, cost weights are additive	Incomplete	Mostly yes
**RxGroups**	Aggregate model (Regression)	Age, Gender, Drug prescriptions	Risk-adjusted reimbursement/compensation systems for Medicare, Rrisk assessment, Efficiency audit of care providers, Calculation of premiums	Boston University, DxCG Inc., USA	Ash, Ellis, Pope et al.[65,72,73,74]	Prospective	No	155 (aggregated to 17 ARCs)	Hierarchical, additive weights for drugs of different hierarchies	Complete	
**RxRisk**	Aggregate model (Regression)	Age, Gender, Drug prescriptions for certain conditions	Risk assessment, Evaluation of severity	Center of Health Studies, Group Health Cooperative of Puget Sound, Seattle, WA, USA	Fishman [75,76]; von Korff [70,71]	Prospective	No	60	Additive weights for drugs of different categories	Complete	
**PCG**	Full hierarchy as cell approach	Regularly outpatient prescriptions of common drugs for chronic conditions	Risk structure compensation system for statutory health insurance in the Netherlands	Erasmus University, Rotterdam, The Netherlands	Lamers & van Kliet [77,78]	Prospective	No	13/23	Original: only the most cost-intense PCG considered; Since 2008: Individual weights are additive	Incomplete	Yes
**PCG+DCG**		Outpatient and inpatient prescriptions					Yes	12			
**DxCG Rx Groups**	Aggregate model (Regression)	Age, Gender, Drug prescriptions, Diagnoses	Prediction of future health costs, Development of a comprehensive Rx classification	DxCG, Inc., MedStat Market Scan	Zhao et al.[66,79]	Prospective	Yes	127(current expansion: 118)	Diagnoses of diverse categories are taken into account	Complete	Mostly yes

The first classification model for drug information was developed in the US in the early 1990s. It has been published as “Chronic Disease Score (CDS)” [70,71]. The target (predicted) variables of the CDS were total costs, outpatient costs and number of doctor’s visits in the following year. In multimorbid patients each identified class of agents contributed to an individual CDS [58].

Based on a data set of approximately 1.9 million Medicaid-insured from four US states, the **Medicaid Rx** model has been developed in 2000 as a further development of the CDS system [80]. Medicaid Rx is a model that may be used to adjust capitated payments to health plans that enroll Medicaid beneficiaries. The Medicaid Rx model includes readily available demographic and pharmacy use data. 48 different diagnostic categories are formed, which mainly include chronic diseases [58]. The calculations of the categories take into account all of the drugs that may be prescribed for each of the 48 disease groups. In addition to its base rate for age and gender an insured person will eventually be allocated with up to 48 surcharges, depending on the drugs prescribed [55]. The Medicaid Rx model recognises the increased medical cost when multiple conditions are identified [80].

The **RxGroup** method was developed in 2001 and has many similarities to the Diagnostic Cost Groups (DCG)/Hierarchical Condition Categories (HCC) model. As base of this model (version 2.0) serve 155 Rx groups, that are compressed to 17 Aggregated Rx Categories (ARCs) and to which approximately 76,000 drugs are assigned. These 17 ARCs are then grouped into a hierarchy according to the severity of the disease expressed by the prescribed drug.[66,79] Within the hierarchy any insured person may only be listed once. The overall compensation per insured is calculated like in most other models: A base rate is paid depending on age and gender. Individual supplements are based on the specific RxGroups [55].

The **RxGroup +IPHCC** model describes a classification model that is solely focused on the predicted cost, but not related to the clinical progression of a disease. This model was developed by DxCG and describes a further development of the described RxGroup. However, this model is only used by a minority of users, since it needs more data to calculate risk adjusted payments. In addition to the information on the use of medications the number and type of inpatient diagnoses is also a necessity. This specific development stage of the RxGroup model includes a combination with the Hierarchical Condition Categories Inpatient. Thus, it takes into account the risk factors age, gender, reduced earning capacity (limited incapacity) status, inpatient diagnoses and outpatient prescriptions to forecast the healthcare costs of the insured more accurately. Considering prescriptions the patients are then assigned to RxGroups, that present pharma-based morbidity groups, as well as to surcharges. Based on the inpatient diagnoses patients are assigned to Inpatient Hierarchical Condition Categories (IPHCCs) and specific risk surcharges [2]. Overall, the model uses 155 different groups, which are combined to 17 hierarchically arranged in aggregates. In total approximately 76,000 drugs are assigned to the 155 groups. Each group comprises drugs for the treatment of a certain condition. Nevertheless, several surcharges are granted for prescriptions from several different RxGroups of drugs [55,81].

This **RxRisk** method was developed in the United States between 1999 and 2000 by Fishman et al. [75,76] as a further development of the CDS-PharmaGrouper by Clark, Von Korff et al. [70,71]. Based on the National Drug Codes of the US Food and Drug Administration the model presents a regression approach for the calculation of the total expenses of the insured. The basis for the calculation is an equation of an age-and gender-related base rate plus any surcharges, if the insured were prescribed a drug belonging to one of the RxRisk classes. The number of RxRisk classes is 60, with chronic illnesses being at the centre [55].

The model of **Pharmacy-based Cost Groups** (PCG) was developed at the Erasmus University of Rotterdam, The Netherlands. Since 2002 this model is amongst others used in the Dutch Healthcare System [58] to perform the risk structure compensation between health insurance funds [55,82].

As in the Medicaid Rx model this model is based on a further development of the CDS model [70]. PCG is focused on prescriptions of drugs that cause comorbidities and high costs in the following year. Reasons for the development of this model can be found in studies on the relationship between suffering from a chronic disease and the use of certain drugs. These studies revealed 28 chronic diseases that supported the assumption according to the appropriate drugs prescribed. To further develop the PCD-method the 28 diagnoses were eventually reduced to the 13 most costly [55].

Mental illnesses were not considered in PCG. In order to be assigned to a PCG a beneficiary needs to have a prescription of the drug of at least 181 Daily Defined Doses in the previous year [55].

The **PCG+DCG** Model was also developed at the Erasmus University of Rotterdam and presents an expansion of the PCG-Model by DCGs [82]. Unlike the PCG it also takes inpatient drug prescriptions into consideration.

In a later modification of this method, the PCGs of cancer and gastrointestinal disorders have been removed. In contrast, neuromuscular diseases, e.g. multiple sclerosis, have been included in the model, resulting in 12 PCGs. Besides the Netherlands, Belgium has decided to use the PCG+DCG method for its risk structure compensation, beginning in 2005 [55].

The **DxCG Rx groups** were developed by Zhao et al. [66,79]. The development aimed at the setting up of a comprehensive Rx classification that encompasses all prescription drugs an individual takes over a fixed period. In order to develop an adjustment model, 58,000 National Drug Codes were assigned “into 127 mutually exclusive categories, called RxGroups” [66]. In a second step these RxGroups were grouped “into 18 aggregated Rx categories (ARC)” [66]. Each ARC symbolizes a major organ system with which an agent interacts or has its primary pharmacologic activity [66]. Within the model, the RxGroups are brought into hierarchies. A patient is assigned only to the category highest in hierarchy, marking the worst condition [58].

The DxCG RxGroups are designed to be an all-encounter model. Therefore both diagnoses coded during outpatient and inpatient encounters were mapped into clinically condition categories. Moreover the model contains markers for age or gender and individuals with several diagnoses are granted surcharges from several categories [66].

#### Risk adjustment methods based on diagnostic information

Diagnostic information is the most direct indicator for the health status of an individual. An advantage of the use of diagnostic data is its high prognostic value [55]. Morbidity is directly described instead of being operationalised by treatment and procedures. However, the disadvantage of diagnostic data is its potential for upcoding [58]. Table 2 gives an overview of available methods that are also briefly described in the following.

**Table 2:** Risk adjustment methods based on diagnostic information.

Method	Cell Approach vs. Regression Model	Risk Factors used	Development Objective	Developing Institution	Developed by (primary author)	Calculation	All-encounter Model	No. of Groups	Comorbidities included	Grouping	Clinical meaningful/interpretable

**ACG/ADG/ADG-PM**	ACGs: cells approach, ADG-Hosdom: aggregate model (Regression)	Diagnoses, Age, Gender, Birthweight, Delivery	Evaluation of severity, Reimbursement/compensation of institutions	Johns Hopkins University, Baltimore, MD, USA	Starfield [83]; Weiner [44,45]; Anderson	Concurrent	ACG: yes ADG: no	ACG: 93 ACG-PM: 236	ACGs are based on combination of diseases	ACG/-PM: complete ADG: incomplete	ACG: no
**HCC**	Aggregate model (Regression)	Diagnoses, Age, Gender	Risk-adjusted reimbursement/compensation systems, Rrisk assessment	Boston University, USA	Ash, Ellis et al.	Prospective	Yes	184	Hierarchical, additive weights for non-related diseases	Complete	Yes
**Rx HCC**	Aggregate model (Regression)	Inpatient + outpatient diagnoses, Drug prescriptions	Calculation of individual risk scores	Medicare Part D, USA		Prospective	Yes	197	Hierarchical, additive weights	Complete	
**CMS-HCC**	Aggregate model (Regression)	Inpatient + outpatient diagnoses	Calculation of individual risk scores	Medicare Part C, USA	Pope et al. [74]	Prospective	Yes	70	Hierarchical, additive weights	Incomplete	
**DCG/DCG-HCC/PIP-DCG**	DCG: complete hierarchy as cell approach, DCG-HCC: Aggregate model (Regression)	DCG/HCC: Inpatient or outpatient and inpatient diagnoses	Risk-adjusted reimbursement/compensation systems for Medicare, Risk assessment, Efficiency audit of care providers, Calculation of premiums	Boston University, DxCG Inc., USA	Ash, Ellis, Pope et al. [65,72,73,74]	Prospective	Original-DCG: no DCH-HCC: yes PIP-DCG: no	DCG: 13 DCG-HCC: 132 PIP-DCG: 10	DCG: individual is assigned to most costly group HCC: hierarchical, additive cost weights	Complete	DCG: no
**PCG+DCG**	Full hierarchy as cell approach	Inpatient + outpatient diagnoses, Drug prescriptions, Age, Gender, Procedures, Region, Reason for insurance	Risk structure compensation system for statutory health insurance in the Netherlands	Erasmus University, Rotterdam, The Netherlands	Lamers & van Kliet [77,78]	Prospective	Yes	12	Original: only the most cost-intense PCG considered; Since 2008: Individual weights of diseases are additive	Incomplete	Yes
**CD-RISC**	Aggregate model (Regression)	Inpatient + outpatient diagnoses, Age, Gender, Medicaid- eligibility, degree of disability	Reimbursement/compensation, Development of risk-adjusted equalization payments,	Rand Cooperation, Santa Monica, CA, USA	Carter; Bell; Dubois [84,85]; Goldberg; Keeler; McAlearny; Post; Rumpel	Prospective	Yes	215	Hierarchical, Individual can be assigned to several hierarchies	Complete	
**CDPS- Rx**	Aggregate model (Regression)	Inpatient + outpatient diagnoses for chronic diseases and disabilities, Age, Gender,	Reimbursement/compensation	University of California, San Diego, CA, USA	Gilmer, Kronick, Dreifuß, Lee [86,87]	Prospective	Yes	19	Considered, Individual can be assigned to more than one group	Complete	
**CRG/CRxG**	Aggregate model (Regression)	Inpatient + outpatient diagnoses, Age, Gender, Procedures, Drug prescriptions	Development of risk-adjusted equalization payments, Monitoring	3M Health Information Systems, Wallingford, CT, USA; US Department of Commerce	Goldfield; Averill et al.	Prospective	Yes	1081	CRGs are based on combination of diseases, Individual is assigned to one of 9 health states	Complete	
**AAPCC**	Cell approach	Age, Gender, Medicaid status, Nursing home status, Employment status + coverage	Compensation of Medicare + choice organizations	HCFA		Prospective	No	122	Not taken into account	Incomplete	No
**ERG**	Aggregate model (Regression)	Diagnoses, Age, Gender, Procedures, Drug prescriptions	Risk assessment, Efficiency audit of care providers, Calculation of premiums	Symmetry Health Data Systems, Inc., Phoenix, AZ, USA	Dunn et al.	Prospective	Yes	120	Individual can be assigned to more than one group	Complete	
**HMG**	Aggregate model (Regression)	Age, Gender, Disability pensioner, Inpatient and outpatient diagnoses (rarely prescription)	Risk structure compensation system for statutory health insurance in Germany	German healthcare act (GKV-WSG)	German healthcare act (GKV-WSG)	Prospective	Yes	178	Individual can be assigned to more than one HMG	Incomplete	
**SQLape**	Predominant diagnoses and procedures: Cell approach; Other diagnoses: Regression	Age, Gender, Inpatient Operations and diagnoses	Prognosis of hospital costs, Development of hospital quality indicators	University of Lausanne, CH	Yves Eggli [88]	Prospective	No	360, Mapping into 17 groups available	Considered, except for predominant diagnoses and operations	Complete	Yes, rough classification
**GRAM**	Aggregate model (Regression)	Diagnoses, Age, Gender	Prognosis of costs for Managed-Care beneficiaries	Kaiser Permanente, USA	Hornbrook, Fishman [75,89]	Prospective	Yes	118/93	Not considered, only highest-ranking diagnosis	Complete	

The **Adjusted Clinical Groups** (ACGs) system was developed in the early 1970s by Starfield et al. [90]. ACG measures the morbidity burden of patient populations based on disease patterns, age and gender [91]. It estimates individual health status and risk for health service use over a defined time interval (one year) [32]. With the help of this model changes over time can be described [34].

Essentially, the ACG assignment process involves four steps. First, the insured persons are grouped into insurance groups based on the International Classification of Diseases (ICD). The ICD diagnoses were compressed to 32 Aggregated Diagnostic Groups (ADGs, sometimes referred to as ADO) [32]. ADGs “aim to be similar in terms of severity and likelihood of persistence of the health condition treated over a relevant period of time” [10]. Hence, each ADG is a cluster of similar conditions (diagnoses) based on their expected impact on health services resource consumption. Patients are then classified into one ACG based on age, gender and constellation of ADGs [32]. An individual can have multiple ADGs [10]. ADGs are then collapsed into 12 major groupings, so-called collapsed ADGs (CADGs). The third step is to collapse these major groupings into 23 commonly occurring categories or patterns, which are referred to as MACs. Assignment to a MAC is unique [10,90]. Originally only the risk factors age, gender and outpatient diagnoses of insured persons were taken into account in the ACG method. Later on inpatient diagnoses were integrated in the calculation [55].

Moreover, lately MACs were further sub-divided, resulting in “53 mutually exclusive ACGs” [10]. As some of the ACGs are further “split into sub-groups a total of 93 ultimate categories”[10] are achieved, allowing for a more accurate assignment of diagnoses [10]. Each outpatient and inpatient diagnosis of an insured person is classified in exactly one ADG.[92] Thus, depending on the type and number of diagnoses, an insured person can be assigned to none, one or more groups. Each ADG is attached a capitation fee [55]. The ACG system has been refined since its development and today there are several variants of ACG model used as method of risk adjustment [10].

The **ADG-Hosdom method** was developed in the 1990s. In contrast to the ACG model, Ambulatory Diagnostic Groups (ADG) form the base. In addition to outpatient diagnoses inpatient parameters are also taken into account. These are called Hosdom parameters, a kind of marker of hospitalization. A positive aspect of this approach is the fact that diagnoses with a high probability of hospitalisation are given a higher weighting, regardless of whether it actually results in an admission to hospital or not [55,57].

In 2003 a cell approach emerged in addition to the existing surcharge approach. The **ACG Predictive Model** (ACG-PM-Model) takes account of the factors age, gender, ACGs, 74 Expanded Diagnosis Clusters (EDCs), and optionally five pharmaceutical expenditure variables as well as the Hosdom parameters. Unlike the ACG-model the ACG-PM forms three categories which differ in their respective expected cost level. The ICD-9 diagnoses are accumulated to 236 EDCs based on medical considerations. An insured person can have several EDCs of a disease group if the appropriate diagnoses are made within one year.

One problem is inherent in the model: The ACG-PM leads to the fact that many diagnoses are counted twice, since they are considered in the ADGs and hence the ACGS, as well as in the EDCs. Moreover, age and gender are taken into account twice, since they are also taken into account in the formation of ACGs already [55,57].

The model of the **Diagnostic Cost Groups** (DCGs) was originally based on the inpatient diagnoses that were recorded in a base year. DCGs were developed by Ash and Ellis at Boston University and Iezzoni at Harvard Medical School in the 1980s [10]. The objective is to estimate the cost of healthcare and to predict the coming period. Similar to the ACG method this model is a cell approach, where every beneficiary is assigned to exactly one insured group. The DCG method condensed the original 14,000 ICD diagnoses to 78 diagnostic subgroups based on medical criteria [93]. Another compression to ten DCGs is based on the average cost of care. The beneficiaries are hence assigned to one DCG according to their diagnoses, age and gender. Care providers do not receive additional payments for multimorbid patients. Therefore, the incentive to select patients is inherent in this model, since the extra effort for treatment is not rewarded financially [55,94]. As with ACGs, the diagnostic cost groups have been refined significantly since their early development. Today the DCG classification mainly includes two major variants, encompassing classification systems for both single condition (PIP-DCG) as well as for multi-condition (DCG/HCC) [10].

The **Principal Inpatient Diagnostic Cost Groups** (PIP-DCGs) system “classifies beneficiaries into 1 of 10 groups, based on the principal inpatient diagnoses they received during hospitalisations in the 6- to 18-month period before the payment period” [95].

This method is a variant of the DCG-model and uses the simplified surcharge approach. Care providers receive a small base rate based on 24 groups defined by age and gender of the beneficiaries. In addition, care providers receive an additional amount depending on whether the insured person was hospitalised in the previous year or not. According to their primary inpatient diagnosis the beneficiaries with a hospital admission are assigned to one of 86 groups that represent influential and costly diseases, which in most cases require a hospital stay and have a high probability of causing high healthcare costs in the future [55,93].

The **Hierarchical Condition Categories** (HCC models) have been developed at the Boston University, mainly by Ash, Ellis and colleagues. Its calculation is prospective. In a first step the more than 14,000 ICD-9 or ICD-10 codes were assigned to 804 DxGroups (Version 6 of the model). The main criteria for the assignment are the medical condition and similarity of diagnoses. For this reason DxGroups also encompass acute medical conditions. DxGroups are further compressed in a second step, based on clinical indicators as well as the expected extent of care. The resulting groups are called “Condition Categories” (CC).

The final step of the model development includes an implementation of hierarchies among the CCs. The Condition Categories are hierarchised according to nature of the disease and severity. Hence, CCs are both clinically- and cost-similar. The HCC-model can therefore be seen as a complete grouper [58,74]. A “person is only coded for the most severe manifestation among related diseases” [74]. Even though HCCs represent hierarchies of related disease categories, HCCs accumulate for unrelated diseases. The HCC model recognises the interaction between certain disease combinations and comorbidities [74].

In general, supporters of the HCC model state, that the categories are clinical meaningful. This makes it easy to interpret in clinical routine by physicians. Moreover, the model can easily be implemented in management programmes, such as Integrated Care programmes. The classification is based on all available diagnoses and diseases as well as transparent criteria of grouping. In terms of the calculation of cost weights in the further process of risk adjusting the clinical consistency is explicitly considered [58].

The DCG method has been modified by the original authors due to accruing criticism by considering the outpatient utilisation in addition to the inpatient utilisation. Moreover, the use of clinical patient data was refined. In contrast to the DCG method, **Hierarchical Condition Categorie**s include the multi-morbidity of the beneficiaries in the risk adjustment, since all diagnoses are incorporated in the calculation. A multimorbid beneficiary is thus assigned with multiple HCC groups and not just one group that includes several weighted diagnoses as in the ACG model. Consequently, a care provider receives lower surcharges for the treatment of a diseased lung cancer patient than for a patient suffering from lung cancer, diabetes and atherosclerosis. Hence, the DCG/HCC can be called a “multiple-condition model” since it “recognizes the cumulative effect of multiple conditions in predicting total medical expenditures” [96]. The US Medicare used this all-encounter risk adjustment mechanism since 2004 [65].

Beginning in January 2004 the Center for Medicare & Medicaid Services started moving “to the **CMS Hierarchical Coexisting Conditions** (CMS-HCC) model, which incorporates inpatient and outpatient diagnostic data” [95]. The risk adjustment was phased in over a seven-year period, ending in 2011 [95].

The CMS-HCC model is basically used for the Medicaid programme Part C to calculate the individual risk score of a beneficiary [58]. Its calculation is prospective. The CMS-HCC model groups the ICD-9-CM codes into 178 disease groups. Out of these groups the 70 diseases that are occasioning most costs and are therefore most predictive of future costs are identified. These 70 disease groups are included in the final 2005 payment model [97]. Pope et al. (2004) discuss the primary criteria for grouping diseases together and for deciding on which diseases comprise the final model [74].

Like the CMS-HCC model, the **RxHCC Diagnostic Classification System** groups the ICD-9-CM codes. However, the RxHCC uses 197 condition categories, also called RxCCs. The RxCCs describe major diseases and are broadly organised into body systems. As in the CMS-HCC model some of the disease groups are clustered in hierarchies. If the drugs for diseases differ from one another, even if the diseases are related, the RxHCCs are not placed in the same hierarchy and remain additive. Conditions not in the same hierarchy contribute independently to the total prediction. After the hierarchies are imposed, the RxCCs become RxHCCs [97]. The RxHCC Model is basically used in the risk adjustment mechanism of Medicare Part D to calculate the individual risk score of a beneficiary [58].

The morbidity-based classification system of **CD-RISC** was initially developed by a US research facility to compare the case mix in physician practices [57]. CD-RISC combines three different types of models. On the one hand is a purely prospective model that takes into account outpatient and inpatient diagnoses. Furthermore, it is a partially prospective model, since it considers the diagnoses from the previous year and on the other hand also the procedures for the current year to provide predictions about the performance issues and costs of the insured. Third, the CD-RISC method includes a retrospective model of risk classification, which takes most of the diagnoses into account, as many acute illnesses are associated with a rather meaningless amount of expenses in the following year in prospective methods. Basis for the classification of morbidity in this model are the ICD-9-CM diagnoses, which are aggregated to 215 diagnostic groups, differing according to the severity of the disease and the age of the insured. The aggregation is based on the expected duration and type of the medical services provided, and the pathophysiology. This combination of diagnostic group, severity and age structure is assigned with 19 body systems. Each of these body systems are again assigned with four up to 34 states, which are sorted into a hierarchy of cost. Every insured person shall be attributed to the combination of condition, severity and age that marks the highest-ranking condition and thus results in the highest cost. When the individual base rate of age and gender is added with these surcharges, the standard costs of an insured emerges. So far, this model for the classification of the insured has not been practically implemented in the wide range [57].

The **Chronic Illness and Disability Payment System** (CDPS) has many similarities with the CD-RISC and the DCG/HCC model. CDPS Rx is a diagnostic classification system that Medicaid programmes can use to make health-based capitated payments for eligible persons of the Temporary Assistance for Needy Families (TANF)-programme and disabled Medicaid beneficiaries. Within the model both inpatient and outpatient diagnoses are assigned to one of 56 categories, which in turn are subsumed to 19 main groups. These 19 main groups are based on body systems or diagnostic types and consist of one to five sub-groups, which are ordered in a hierarchy according to related costs and/or expenses [86,87]. At maximum, each insured person can be attributed to each of these 19 groups once. The total amount of the expenses for an insured person eventually results from the sum of the base rate for age and gender as well as the maximum of 19 surcharges [57,98].

The **CRG**-method (**Clinical Risk Groups**) was developed by the US Department of Commerce and is based on inpatient and outpatient diagnoses as well as procedures to determine the payments for insured of all ages. Here, the insured are assigned to one of up to 1081 cells in a 3-phase sequence. These cells are called Clinical Risk Groups (CRG). To do so, information about age, gender, ICD-9-diagnoses, procedures, type and sector affiliation of the care provider as well as date and frequency of treatment are obtained. In order to be considered within the adjustment, outpatient diagnoses need to be made on at least to independent days. In the first step 534 Episode Disease Categories (EDCs) are formed, which are assigned with the diagnoses of the insured. Six types of EDCs, which are arranged hierarchically, can be differenced. The 534 EDCs are in turn allocated to 37 organ-related Major Diagnostic Categories (MDCs). In addition to the diagnoses, the treatments and procedures are assigned to one of 605 Episode Procedure Categories (EPCs), 63 of whom also lead to inclusion in an EDC. In a second phase of adjustment a Primary Chronic Disease (PCD) is defined. Therefore the most important EDC is selected within each of the 37 MCDs, if corresponding EDCs exist with the patient. The basis for the selection is the presented hierarchy of EDCs. In phase 3, the insured are assigned to one of the 1081 CRGs. This assignment is based on the type, number, and the associated severity of the PCDs. The insured person is assigned to one of nine states, ranging from “healthy” to “catastrophic”. These nine states are assigned with a total of 271 base CRGs. In 7 of the 9 states a distinction is made between degrees of severity. This results in 1081 CRGs. The grouping algorithm ensures that etiologically/pathogenetically heterogeneous cells arise [57]. Finally, benefit payments for each of the 1081 CRGs are calculated [99].

The CRxGroup-method was developed on the basis of the CRG model. Next to the risk factors described above this new method takes also prescriptions of drugs into consideration, regardless of whether the drugs were taken once or are prescribed permanently. The process of risk adjustment and calculation of benefit payments is otherwise identically with the procedure of CRGs [57].

The **Adjusted Average Per Capita Cost** (AAPCC) method was used by Healthcare Financing Administration (HCFA) to compensate Medicare+ Choice organisations for care of Medicare patients [100]. In 1985 Medicare first implemented the Adjusted Average Per Capita Costs (AAPCC) payment methodology [101]. AAPCC presents a county-level estimate of the average cost incurred by Medicare for each beneficiary in the fee-for-service system. The AAPCC is made up of 122 different rate cells; 120 of them are factored for age, gender, Medicaid eligibility, institutional status, and whether a person has both part A and part B of Medicare. Separate AAPCCs are calculated at the county level for Part A services and Part B services for the aged, disabled, and people with end stage renal disease. Medicare pays risk plans by applying adjustment factors to 95 percent of the Part A and Part B AAPCCs. The adjustment factors reflect differences in Medicare per capita fee-for-service spending. Adjustments are made so that the AAPCC represents the level of spending that would occur if each county contained the same mix of beneficiaries.

Criticism of this model is rather high. Critics say that “risk adjustment models that consider total medical costs are much more accurate in their ability to predict future medical costs than the current AAPCC methodology.” [100]. According to them, AAPCC rates do not measure actual health risk for the portion of the Medicare-eligible population that consumes the most healthcare services. Hence, many Health Maintenance Organizations (HMOs) have stopped its use in Medicare+ Choice programmes and switched to another method of risk adjustment [100]. AAPCC is currently rarely used as mechanism of risk adjustment.

As most other methods of risk adjustment the **Episode Risk Groups** (ERG)-surcharge model was also developed in the US in 2001. The prospective model differs from other approaches mostly by the fact that its algorithm of grouping is based on time episodes. In addition to the risk factors age, gender, diagnoses, and procedures, drug prescriptions are also taken into consideration. Here, the beneficiary only receives bonuses, if in a three-stage process of calculation at least one of the 120 ERGs turns out to be significant. Surcharges are calculated using a regression model.

In the first phase, episode clusters are formed that contain information about individual cases, utilisation such as medical indications and contacts (“anchor records”) as well as additional services (“ancillary records”, e.g. laboratory tests). The focus on individual cases marks a differentiation from conventional models of risk adjustment. In the second step the clusters are assigned to approximately 600 episode treatment groups (ETGs). The assignment is based on the ICD-9-CM diagnoses, procedures, and a pharmaceutical grouper. The pharmaceutical grouper is used to sort the prescribed drugs in a therapeutic classification model. The third and final stage includes an aggregation of the ETGs to 120 ERGs [55].

**Figure 2:**
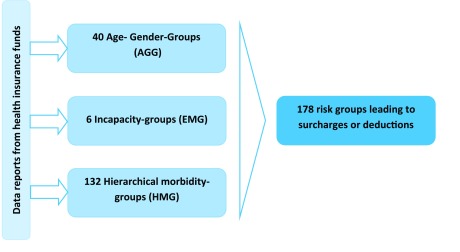
German morbidity-oriented risk structure compensation scheme [104].

As many other mechanisms worldwide, the German risk structure compensation scheme is a prospective model, too. The “morbidity-oriented risk structure compensation scheme” [102] was introduced together with the health fund in 2009. Since then the German risk structure compensation, the so-called “Morbi-RSA”, is a prospective regression approach in which the compensation of care providers is retrospective [58]. The following factors are incorporated in the mechanism: Age, gender, hierarchical morbidity groups, groups for reduced earning capacity and other groups of insures differentiated by their entitlement for sick-pay. Moreover, a special group of so-called “insured abroad” is formed. These beneficiaries are Germans currently not living in Germany while being insured in the SHI [58]. At first, each health insurance receives a basic fee for each insured person in the amount of average per capita expenditure of the SHI (2012: 209, 48 €/month) [103]. Over time this base rate is adapted through a system of surcharges/premiums and deductions. In addition to the existing features of the risk adjustment – age, gender, and eligibility for disability/limited incapacity pension – the disease burden of a health insurance should be taken into account measured on the basis of 80 selected diseases. The selection of the 80 diseases/affections was based on five main criteria: closely definable, “cost-intensive and chronic”, severity/severe course, cost-intense (if not severe or chronic), relevance for healthcare system and cost-relevant for the SHIs [58]. Additionally, supplements are provided if the insured person receives a disability pension. The morbidity-based risk structure compensation thus consists of three pillars: the surcharges and deductions for age and gender, the surcharges for disability pensioners and the disease-based allowances. For the classification into a disease group 132 morbidity groups are used. Hence the 2012 version has 178 risk groups (see Figure 2) [104].

The cost weights for the surcharges and deductions are calculated using a non-negative, hierarchical and weighted regression [58].

With the help of the hierarchy the most serious manifestation of a disease in each case should be determined. And exclusively for this the allowance is granted. In 2012 the surcharges and deductions ranged from 15€ to 25,178.86€ per month [103]. Since not all ICD-diagnoses are grouped into HMGs the German risk structure compensation mechanism is an incomplete grouper [58]. In practice, any relevant inpatient diagnosis leads to an assignment of the beneficiary to a HMG and thus to a surcharge. However, for some diseases additional proof of appropriate therapy is necessary [103,105].

The **SQLape** classification model was developed by Eggli in Switzerland and is based on a combination of procedures and inpatient (hospital) diagnoses. The model is used since 2005. The main aim is the prognosis of hospital costs and the development of hospital quality indicators. However, since its development this method has also successfully been used as risk structure compensation model [58].

The process of classification consists of several steps.

1. A decision is made on whether a patient can be classified based on one or more operation(s) and/or diagnoses/affections. Therefore so-called predominant operations are analysed. If the patient underwent this (e.g., Coronary artery bypass grafting) during his or her hospital stay the patient is classified based on that. All other diagnoses/procedures are not taken into account.
2. If no predominant operation is present, the presence of a so-called predominant diagnosis (e.g., heart failure) is checked. If the patient has such a diagnosis the classification is based on that. Again, all other diagnoses/procedures are not further considered.
3. If the patient has neither a predominant operation nor a predominant diagnosis, the classification is based on other indicators, such as significant diagnoses or other procedures. If diverse SQLape categories are applicable to the patient, the patient is classified into all categories [58].

Inpatients are allocated to one or several affections or operations. Each category is defined by inclusion and exclusion criteria. Inclusion criteria are “based on the Swiss surgical intervention nomenclature (CHOP-XI, a Swiss adaptation of the ICD-9-CM) for operations and on the International classification of diseases (ICD-10) for affections, including optional additional codes (German adaptation of ICD-10)” [88]. All together SQLape defines 180 affections and 180 operations, resulting in 360 available categories. In order to be used in risk adjustment the 360 categories need to be summarised and compressed to a smaller number of categories. Holly et al. suggest to aggregate 17 medical adjustment categories based on clinical criteria [12]. Proponents of this model point out the fact that SQLape is very easy to interpret in clinical terms. Moreover the model explicitly deals with comorbidities. However, compared to other methods of risk adjustment SQLape has a high data need because of the consideration of operations and procedures [58].

The development of the **Global Risk Assessment Model** (GRAM) method was based on a dataset of one million Kaiser Permanente beneficiaries of all ages. In the process of grouping, the ICD-9-CM diagnoses of the beneficiaries were assigned to 118 Clinical Behavioural Subclasses (CBS) depending on their medical context and the probable treatment patterns. These 118 subclasses of clinical behaviour therapy are classified into 19 main groups (Clinical Behavioural Classes) and three major groups (serious illness, less serious diseases and no diseases). The CBS are hierarchically grouped into five categories based on expected cost intensity. These categories are called Clinical Resource Intensity Groups (CRI) and differ in terms of their costs starting at “extremely high” to “average”. Overall, 93 Clinical Behavioural subclasses have been defined. In this model each beneficiary is only considered with the highest-ranking diagnosis, and thus taken into account only once, regardless of whether there is an additional diagnosis of other CRIs of the same CBS. Hence, a beneficiary can be assigned with up to 93 CBSs [55] The model is currently not actively used in terms of risk adjustment and/or risk structure compensation [2].

#### Risk adjustment with information on (self-reported) health status

The inclusion of socio-economic variables such as income, education, or social class into the risk adjustment is feasible since a number of international studies support an association between morbidity and socioeconomic status. Often not only pure income categories are considered, but also the type of work (employee, employee at management level, workers), educational level and employment status (unemployed, duration of unemployment). In each of these categories a high socio-economic status is associated with a lower health risk. In fact, the inclusion of socio-economic variables into the risk adjustment is implemented in certain countries. The Dutch risk structure compensation for example takes the socio-economic status into account by incorporating accumulated income groups as a countervailing factor since 2008 [67].

The inclusion of such a criterion into the risk adjustment mechanism would slightly increase the explanatory power. In addition, socio-economic status is not vulnerable to manipulation. Moreover, the efficiency of resource use is not affected by socio-economic status, since this is not connected to a particular treatment. However, a problem is the data collection. The health insurers today receive hardly any information about the socio-economic status of their insured [67].

Tools for assessing and measuring health-related quality of life and self-reported health status can also be used in risk prediction and risk adjustment models. In addition to the widely used instruments, like the SF-12 and SF-36 various MCOs in the United States have also developed own instruments. These are usually used to identify individuals with a high health risk and to plan appropriate interventions where necessary. It is questionable to what extent the risk attitude of individual insured allows the prediction of future healthcare costs. However, based on this information interventions can be designed goal-oriented [106].

Some authors point out that the quality of life indicators (HRQL) from the SF 36 do forecast the expected expenditure better than the evaluation of utilisation data (for further information see [107,108]). However, self-reported data have disadvantages: high costs and complex administration. The possible collection of data (self-reported) from insured-surveys for risk adjustment is used mainly for demonstration purposes, the use for risk adjustment of reimbursements of doctors or organizations of providers has not yet gained acceptance [106].

## Discussion and conclusion

The problem of an aging and sicker population is ubiquitous. The demographic change and technological progress are two of the reason for the fact that anywhere in the world health systems face the challenge of an aging population and an increasing number of chronically ill patients as well as multi-morbidity [109]. By their increased utilisation of care delivery systems these insured patients generate most of the costs while potentially paying hardly any contributions as pensioners. The resulting costs need to be covered by healthcare. Integrated Care programmes are concepts that have proven to be an efficient solution to achieve structured health delivery. However, all Integrated Care systems are facing the problem of an effective risk adjustment, in order to deliver efficient services in a long run.

Risk adjustment is considered to be a standard method of epidemiology and used for a variety of purposes. It includes a compensation of different populations in terms of their different disease severity. One of the principal uses of risk adjustment is to set payments for health plans or Integrated Care programmes to reflect expected treatment costs of their insured clients. Because of differences in health status and treatment needs, the cost of health care will vary from person to person [110]. Patients characteristics such as age, gender, severity of disease and possible comorbidities are here eliminated in their magnitude. This should be achieved to best meet the different patient population.

Risk adjustment methods can be distinguished between simple models and those based on diagnoses. The simple models are basically focused on demographic characteristics such as age and gender, which can be easily determined. However, those models have limited explanatory power on expected treatment costs. If at all, these models should be used for insurance companies with high number of insured persons. Diagnosis-based methods of risk adjustment also take into account other risk factors [56].

After the final examination of different methods of risk adjustment it was shown that the methodology used to risk adjust varies, depending in part on healthcare market regulations, the populations served and the source of payments. Moreover, the models differ greatly in terms of their included morbidity indicators. The basic indicators age and gender are included in all but two of the analysed models. Moreover, the diagnoses are integrated in many models. Finally, it cannot be judged which model is the most efficient for an Integrated Care programme. The selection of one specific model should always be based on the consideration of indicators needed as well as the objective of the use and the underlying characteristics of the integrated healthcare system.

By considering the morbidity factors and the related risk adjustment mechanisms risk selection is counteracted, which has a positive effect on the insured population. In addition, payers and providers have to compete against each other in terms of quality and efficiency, which is also recorded positive and affects the insured patients and their treatment.

In general, the findings of this review can be used in the evaluation of integrated/organized healthcare delivery systems and can be integrated into quality- and patient-oriented reimbursement of care provider in the design of healthcare contracts.
